# The first detection of two *Aeromonas* strains in mice of the genus *Apodemus*

**DOI:** 10.1038/s41598-023-31306-3

**Published:** 2023-03-15

**Authors:** Pavel A. Andriyanov, Daria D. Kashina, Elena A. Liskova, Pavel A. Zhurilov, Anastasia I. Tutrina, Svetlana A. Ermolaeva, Olga I. Zakharova, Andrey A. Blokhin

**Affiliations:** Federal Research Center For Virology And Microbiology, Branch in Nizhny Novgorod, 603950 Nizhny Novgorod, Russia

**Keywords:** Microbiology, Bacteriology

## Abstract

*Aeromonas* spp. are gram-negative facultatively anaerobic bacilli recovered mainly from aquatic environments. *Aeromonas* spp. were reported to be associated with infections primarily in aquatic and to a lesser extent in terrestrial animals as well as in humans. Up-to-date little is known about aeromonads associated with wild animals, especially with rodents. This study reported the first isolation and characterization of two *Aeromonas* spp. from internal organs of apparently healthy wild rodents *Apodemus uralensis* and *Apodemus flavicollis* captured in the wild environment in the European part of Russia. Isolates were identified as *A. hydrophila* M-30 and *A. encheleia* M-2 using the multilocus sequence analysis (MLSA) approach. The isolation of the *A. encheleia* from rodents is the first described case. Both strains demonstrated beta-hemolytic activity towards human erythrocytes. Antimicrobial susceptibility testing showed that both *Aeromonas* strains were resistant and intermediate to carbapenems and piperacillin-tazobactam, which was caused by the expression of the genus-specific CphA carbapenemases. *A. hydrophila* M-30 also demonstrated trimethoprim resistant phenotype. This is usually caused by the carriage of the *dfrA* or *dfrB* genes in aeromonads which are frequently associated with integron class I. The latter however was absent in both isolates. Our results expand our understanding of possible aeromonad reservoirs and demonstrate the likelihood of the formation of natural foci of *Aeromonas* infection and a new link in the chain of the spread of antimicrobial resistance as well.

## Introduction

Aeromonads are ubiquitous Gram-negative, facultative anaerobic bacteria. The genus *Aeromonas* includes more than 35 species recovered mainly from aquatic environments and diverse animal species but also clinical settings, and human infection processes. Aeromonads were isolated from different kinds of water including surface, underground, seawater, wastewater, and even bottled water^1–5^. They are thought to be primarily autochthonous to aquatic environments and were also found in a variety of aquatic animals, such as mollusks^6^, sick salmonids^7^, catfish^8^, eels^9–11^, crocodiles^12^. However, they were also isolated from terrestrial animals, for instance, rectal swabs of cats, dogs^13^, and horses' feces^14^.

Aeromonads are known as fish pathogens, first of all, such species as *A. salmonicida* and *A. hydrophila*. The first of them—*A. salmonicida*, in particular, infects salmonids and causes ulcers, furunculosis, and hemorrhagic septicemia, bringing tremendous economic losses to continental and marine aquaculture^15^. Meanwhile, some hypervirulent lineages of *A. hydrophila* are responsible for large-scale persistent outbreaks globally characterized by massive hemorrhage and exophthalmia in warm-water fishes^16^. However, due to frequent misidentification, the certain incidence in fish between these two species is hard to evaluate.

*Aeromonas* species are also known to infect humans. Aeromonads are emerging pathogens that can cause acute gastroenteritis and wound infections and are occasionally involved in life-threatening septicemia in immunocompromised and immunocompetent persons. The most prevalent species that are associated with clinical cases are *A. caviae*, *A. veronii*, *A. dhakensis*, and *A. hydrophila*^17^. The pathogenic potential of these species is different. Comparative analysis performed by P.-L. Chen et al. in 2013 was shown that *A. dhakensis* prevailed in wound infection exposed to environmental water and was more virulent than *A. hydrophila*, exhibiting increased biofilm formation ability and higher cytotoxicity to human fibroblast cells^18^. It was noted that the transmission of virulent *Aeromonas* strains occurs via the fecal–oral route through both indirect and direct contact with contaminated water^19,20^. Thus *Aeromonas* spp. constitute a heterogeneous group, some members of which are clinically important species occurring in humans.

Both clinical and environmental isolates belonging to this genus often possess antibiotics resistance determinants^21–23^. Antimicrobial resistance is one of the main points of the One Health holistic approach that implies the interconnection between the three linked key elements of biosphere well-being: humans, animals, and the environment^24^. The latter has drawn increasing attention because the natural environment can serve as a reservoir of antimicrobial resistance (AMR) of both genes and bacteria itself^25–27^. This is notably true for aquatic environments which are primarily exposed to wastewater from hospitals, clinics, and industry^28^. Being the natural inhabitants of such an environment, aeromonads may acquire various resistant genes and disseminate the latter via horizontal gene transfer^29^.

Wild animals are also of high importance in the One Health concept^30^. They constitute a potential natural AMR and infection reservoir. They also actively participate in the dissemination of bacteria and resistance determinants across different habitats^31,32^. Apart from aquatic animals some *Aeromonas* spp. were isolated from feces of wild terrestrial animals: *A. salmonicida* from red deer (*Cervus elaphus*) and tawny owl (*Strix aluco*); *A. bestiarum* from red squirrel (*Sciurus vulgaris*); *A. eucrenophila* from snake (*Colubridae*), and *A. veronii* from short-toed snake eagle (*Circaetus gallicus*)^33^. Interestingly, almost half of the above isolates were multidrug-resistant and possessed acquired beta-lactamase genes. Taking into account that *Aeromonas*, including their pathogenic strains, are ubiquitous and can transmit and circulate through the food chain from water to various terrestrial animals and humans it may serve as a vehicle for AMR spreading. In this regard, little is known about biological features, such as AMR profile and pathogenic potential of *Aeromonas* spp. isolated from animals inhabiting different wild environments.

In this study, we report two *Aeromonas* strains isolated from the internal organs of apparently healthy rodents captured in a wild environment. We performed genus identification via 16S rRNA gene sequencing and used the MLSA approach for identification at species level. Antimicrobial susceptibility profiles as well as antimicrobial resistance genes detection and analysis were carried out. Additionally, we assessed virulent properties via in vitro hemolytic activity evaluation and detection of major virulence genes via PCR.

## Results

### Sample collection

In total, 37 captures of apparently healthy small mammals (36 belonged to the species of Rodentia order and 1 of Insectivora) were recorded during the month (June 2021). Among the 4 species of small mammals that were captured, the herb field mouse (Apodemus uralensis) represented 40.5% (n = 15), bank vole (*Clethrionomys glareolus*)—27.0% (n = 10), yellow-necked mouse (*Apodemus flavicollis*)—5.4% (n = 2) and common shrews (*Sorex araneus*)—2.7% (n = 1) of total captures. A total of 65 different Gram-negative isolates were received from internal organs of captured small mammals. Only two isolates of them (3.0%) were further identified as *Aeromonas* spp. via 16 s rRNA gene sequencing.

### Isolation and identification of *Aeromonas* strains

The two strains of *Aeromonas* were isolated from the internal organs of captured apparently healthy wild rodents. Strain M-2 was isolated from the spleen of a female Herb field mouse (*Apodemus uralensis*) through the PSB Broth Base and further CIN agar cultivation. It produced small bull’s-eye mucoid colonies on CIN agar, resembling the growth of *Yersinia* spp.

The M-30 strain was isolated from the lungs of a male yellow-necked mouse (*Apodemus flavicollis*) via the BPW broth and further XLD agar cultivation. Microscopic investigations showed the typical Gram-negative bacilli morphology of both strains. Studied strains had catalase and oxidase-positive phenotypes.

**Figure 1:**
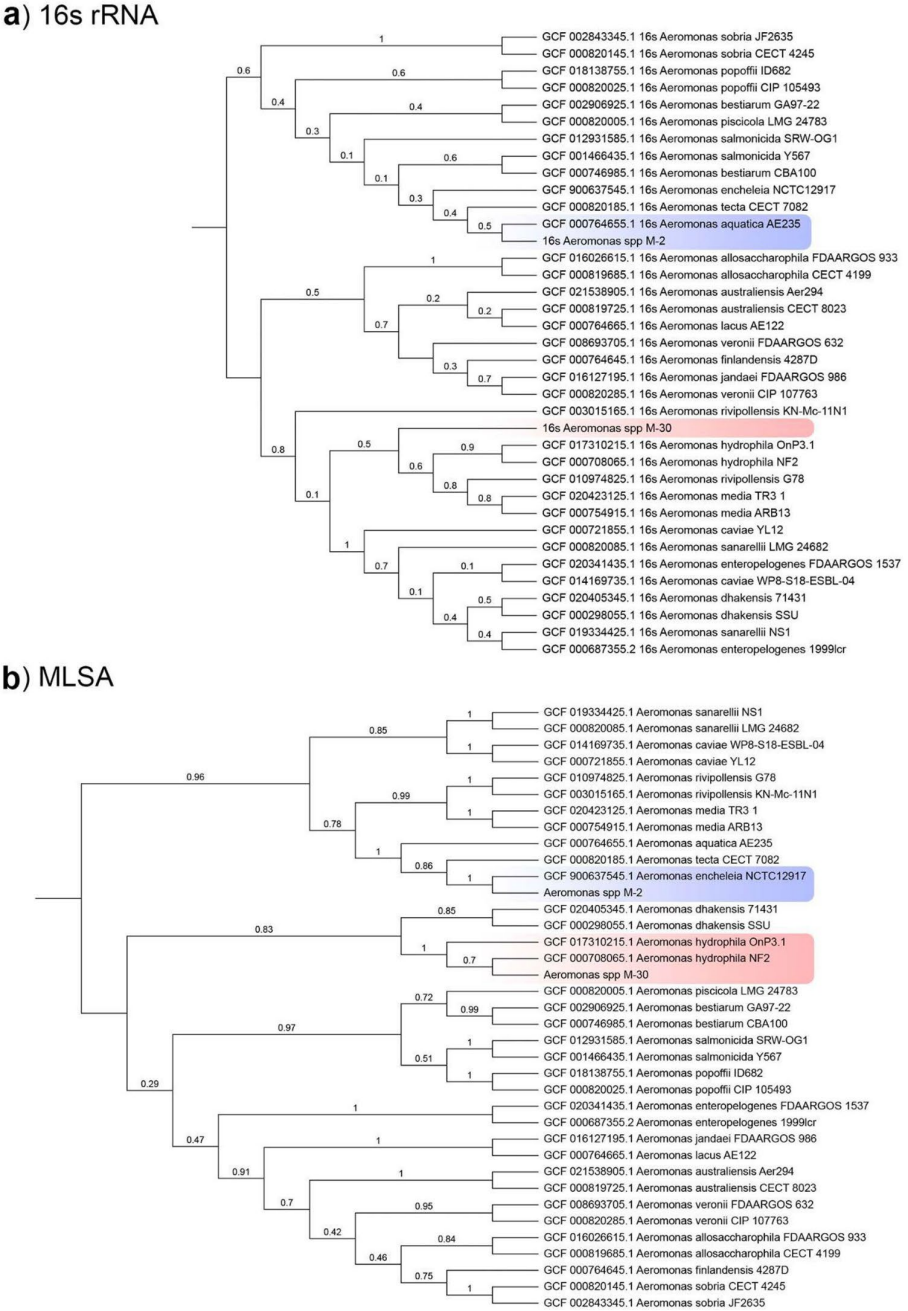
Reconstructed cladograms of M-2, M-30 and 35 *Aeromonas* spp. strains based on: (**a**) 16 s rRNA gene sequence-based cladogram. (**b**) MLSA based cladogram (5 loci). Cladograms were visualized in Interactive Tree Of Life v5 (iTOL)^34^.

We performed initial identification via 16 s rRNA gene sequencing using 27f. and 1492r universal primers. Assembled 16 s rRNA gene consensus was 1367 bp for M-2 isolate and 1412 bp for M-30. The highest value of sequence identity of M-2 consensus was 99.85% with the sequence of the *A. aquatica* AE235(T) and *A*. *encheleia* LMG 16,331(T), 99.36% of identity with the *A. media* CECT 4232(T) was detected for M-30 consensus. The maximum-likelihood tree, based on 16 s rRNA gene sequences (alignment length 1276 bp) of our two and 35 *Aeromonas* spp. strains showed that M-2 clustered together with *A. aquatica* AE235 (GCF 000,764,655.1) while M-30 was in the outgroup position regarding *A. hydrophila-rivipollensis-media*-clade (Fig. 1a).

### *Aeromonas* species identification by the Multilocus sequence analysis (MLSA) approach

It is well known that the *Aeromonas* genus has a complex taxonomy that cannot be resolved only by 16 s rRNA gene sequencing. Therefore, we performed multilocus sequence analysis (MLSA) based on 5 loci (*groL*, *gltA*, *metG*, *ppsA*, and *recA*) from Martino et al. scheme to resolve the issue of the species of our studied strains. The maximum-likelihood cladogram reconstructed from the alignment of concatenated loci (total length 3084 bp) of our and 35 *Aeromonas* spp. strains showed that M-2 strain formed one monophyletic group with *Aeromonas encheleia* NCTC12917 (GCF 900,637,545.1) with a bootstrap of 100 whereas M-30 strain made one phylogenetic group with the *A. hydrophila* strains, where the closest one was NF2 (GCF 000,708,065.1) with 70 value bootstrap. Because MLSA has been offered as a gold standard taxonomic approach to *Aeromonas* species delineation our strains were identified as *A. encheleia* M-2 and *A. hydrophila* M-30 (Fig. 1b).

### Antimicrobial susceptibility testing of *Aeromonas* strains

We estimated the antimicrobial susceptibility of M-2 and M-30 strains to 15 commonly used antimicrobials via the disk-diffusion method following CLSI guidance. Susceptibility patterns as well as the diameter of inhibition zones are provided in Table 1.

**Table 1:** List of antimicrobials used and susceptibility pattern with zone diameter in mm.

Antimicrobial class	Antibiotic	M-2	M-30
Beta-lactams	Piperacillin/tazobactam	14	0
		R	R
	Meropenem	21	18
		I	R
	Imipenem	21	19
		I	R
	Ceftazidime	22	27
		S	S
	Cefepime	28	31
		S	S
	Aztreonam	40	36
		S	S
Aminoglycosides	Gentamicin	22	18
		S	S
	Amikacin	22	18
		S	S
Fluoroquinolones	Enrofloxacin	32	29
		S	S
	Ciprofloxacin	34	36
		S	S
	Levofloxacin	35	34
		S	S
Other	Trimethoprim/sulfamethoxazole	28	24
		S	S
	Tetracycline	27	27
		S	S
	Chloramphenicol	26	30
		S	S
	Trimethoprim	27	12
		S	R

*A. encheleia* M-2 was resistant to piperacillin-tazobactam only and had an intermediate level of resistance (I—susceptible with increased dosing) to both carbapenems tested (meropenem and imipenem). Whereas *A. hydrophila* M-30 was resistant to 4 antimicrobials including the 3 beta-lactams as well as 1 diaminopyrimidine: piperacillin-tazobactam, meropenem, imipenem, and trimethoprim respectively. Both strains were susceptible to other tested beta-lactams: cephalosporins and monobactam; as well as aminoglycosides, fluoroquinolones, tetracycline, chloramphenicol, and trimethoprim-sulfamethoxazole.

### PCR analysis

**Figure 2:**
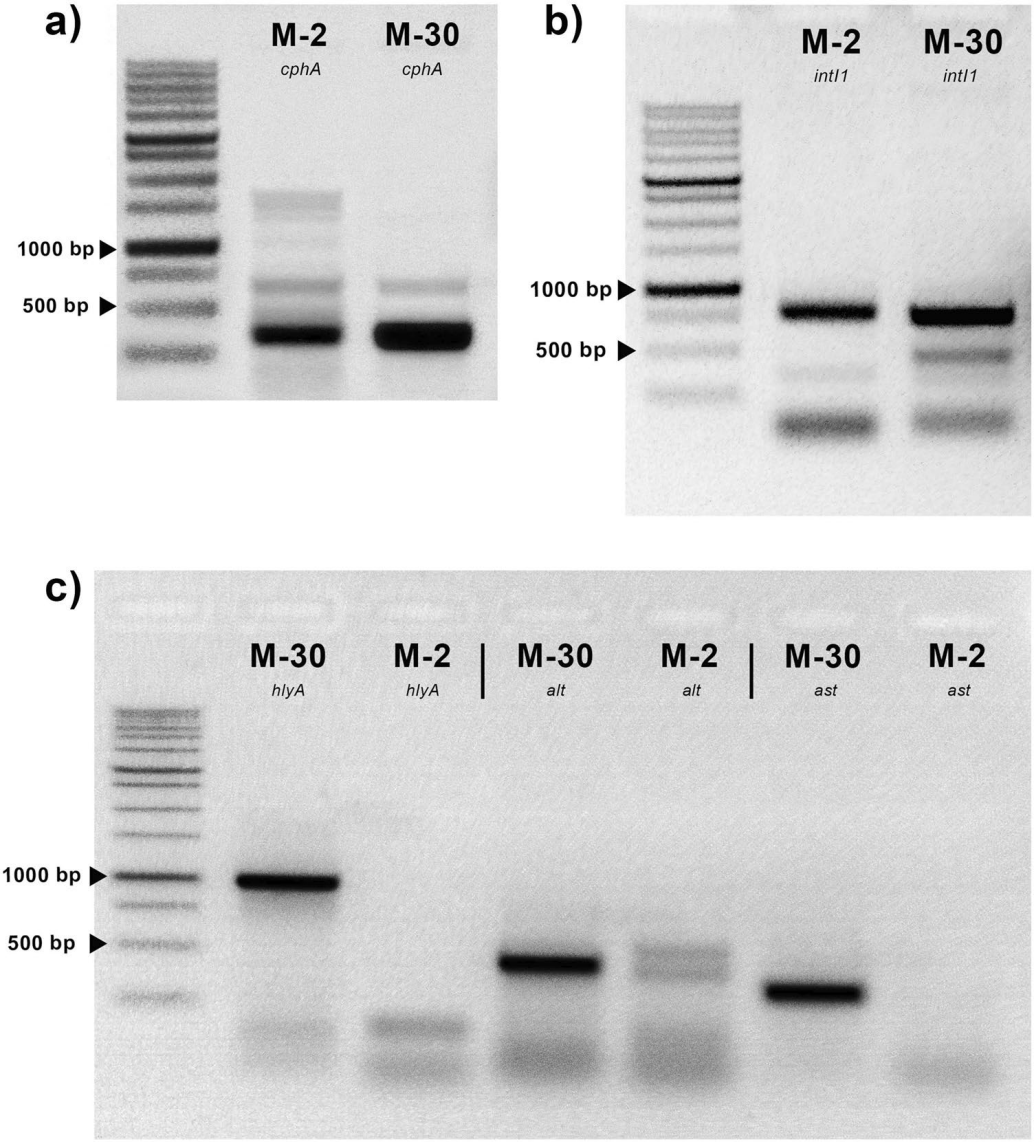
Agarose gel electrophoresis of target amplicons: (**a**) *cphA* gene (346 bp). (**b**) putative *intI1* gene (920 bp). (**c**) major virulence genes—*hlyA* (1079 bp), *alt* (442 bp), *ast* (331 bp).

The presence of *cphA* genes in M-2 and M-30 strains coding for the unique *Aeromonas*-specific beta-lactamase (Fig. 2a, original Fig. S1) was detected through PCR with specific primers, as indicated by a product size of 346 bp.

Using primers for the integrase (*intI1*) gene amplicons with a size of 920 bp were also detected (Fig. 2b, original Fig. S2). Obtained amplicons were sequenced and searched against GeneBank through blastx. *intI1* M-2 amplicon was matched as glyceraldehyde-3-phosphate dehydrogenase, *intI1* M-30 was matched as cytochrome b-b6 domain-containing protein. Putative variable regions were also amplificated: for M-2 strain 1000 bp and 900 bp for M-30. Both amplicons were sequenced: the amplicon of M-2 strain matched as 50S ribosomal protein, and the amplicon of M-30 strain matched as DUF3450 domain-containing protein in blastx search.

The presence of *hlyA* genes was detected in M-30 strain which correlates with their hemolytic activity on 5% blood agar. Other major virulence genes *alt* and *ast* were also detected in M-30 *A*. *hydrophila* via PCR. Meanwhile, M-2 isolate has no detected virulence factors through PCR (Fig. 2c, original Fig. S3).

### Hemolysis assay

**Figure 3:**
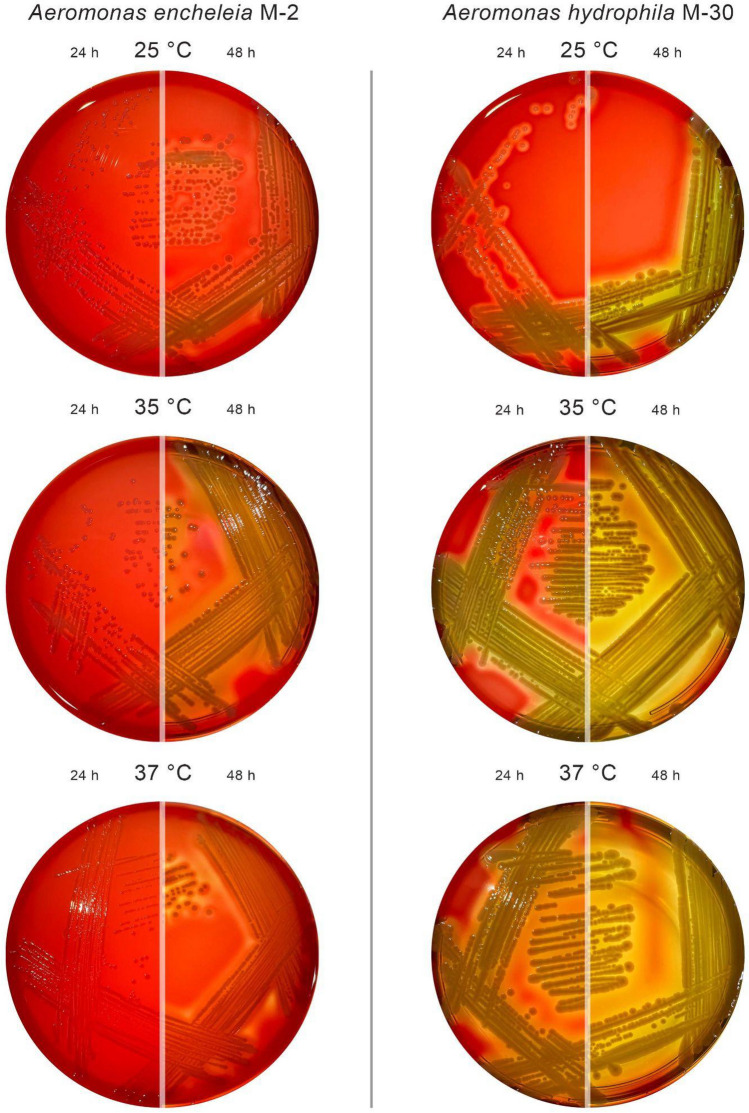
Hemolytic activities of M-2 and M-30 *Aeromonas* strains on 5% human O-blood agar plates.

Hemolysis investigation of both strains was performed at 3 different temperature points: 25 °C, 35 °C, and 37 °C for 24 h and 48 h (Fig. 3). *A. encheleia* M-2 strain showed gamma-hemolysis in all tested temperature conditions after 24 h. However, the beta-hemolysis was observed after 48 h of cultivation at all temperatures, but most clearly at 35 °C. *A. hydrophila* M-30 demonstrated beta-hemolysis after 24 h. Clear transparent zones of 2–4 mm in size were observed around the M-30 colonies when cultivated at all temperature points.

### Virulence factors production

The culture-based method was utilized to detect the production of the following virulence factors: gelatinase (proteases), lecithinase, lipase, and flagellum.

The gelatinase test was positive for *A. hydrophila* M-30 (liquid fraction formation was detected) and negative for *A. encheleia* M-2 (Fig. S4).

Lecithinase activity (formation of precipitation on egg yolk agar) was detected after 48 h of incubation in M-2 strain only (Fig. S5), however on the third day weak precipitate formation was also detected around the M-30 colonies. Lipase activity was negative (iridescent sheen was not detected) in both strains. Protease activity (clear zone formation) was positive in both strains, although visually M-30 had a larger clear zone than M-2 (Fig. S5).

Motility was detected in M-30 only (diffuse growth was observed), while M-2 grew strictly along the inoculation trail (Fig. S6).

## Discussion

Aeromonads are ubiquitous Gram-negative bacteria. They are thought to be primarily autochthonous to aquatic environments. In this study, two strains of *A. encheleia* M-2 and *A. hydrophila* M-30 that were isolated from the internal organs of apparently healthy wild rodents have been identified. We characterized the antimicrobial resistance profile and hemolytic activity as well as analyzed the resistance determinants and the main virulence genes of both strains via PCR approach and with a culture-based method.

Previous studies have reported the isolation of *A. encheleia* strains to occur on the surface as well as in underground water^1^, in European eels^10,35^, and in drinking water supply^35,36^. To the best of our knowledge, the first case of *A. encheleia* isolation from wild rodents is reported here. However, the significance of the detection of *A. encheleia* in the lungs is questionable due to potential aspiration during agony. It is believed that *A. encheleia* are not pathogenic for eels or mice^10^. We were unable to find data on the pathogenicity of *A. encheleia* for humans as well. However, several *Aeromonas* species were found as pathogens in a variety of cold-blooded and warm-blooded animals, including humans^37,38^. Aeromonads can cause serious diseases in humans: acute gastroenteritis, wound infections, and septicemias in immunosuppressed patients^39^.

*A. hydrophila* is predominantly pathogenic to poikilothermy animals, including fish, turtles, snakes, and amphibians^37^. However, *A. hydrophila* can infect humans as well, moreover, Risco et al. reported the isolation of *A. hydrophila* in the respiratory tract of wild boar piglet in Spain with severe purulent pneumonia which defines this bacterium as a zoonotic pathogen that may pose a potential risk to people^40^. Interestingly, we discovered a hemolytic activity possessed by *A. hydrophila* M-30 and *A. encheleia* M-2 to human erythrocytes. Moreover, we detected three major virulence genes in *A. hydrophila* M-30, including hemolysin (*hlyA*), cytotonic heat-labile (*alt*), and heat-stable (*ast*) enterotoxins via PCR. Previous investigations reported that all clinical isolates of *Aeromonas* spp. harbor *hlyA* or *aerA* genes, which encode hemolysin (HlyA) and aerolysin (AerA), respectively. It indicates hemolysins are essential virulence factors participating in the pathogenesis of infections caused by *Aeromonas*^41^. Singh et al. revealed the correlation between beta-hemolytic activity and enterotoxic effect in the rabbit ileal loop infection model independently of aeromonads species designation^42^. *Aeromonas* hemolysins were shown to not only damage the intestinal epithelium but also stimulate chlorine secretion, which indicates that they are the key diarrheal etiological factors^43^. *Aeromonas* spp. may also carry cytotonic heat-labile (*alt*) and heat-stable (*ast*) enterotoxins that are considered to be the major virulence determinants associated with gastroenteritis, especially with diarrhea^44^. The activity of other virulence factors such as gelatinase, lecithinase, lipase, and motility was tested via a culture-based method. Interestingly, only the A. hydrophyla M-30 strain was positive for gelatinase and motility at 37 °C and negative for lecithinase. However, a weak precipitation zone was observed on the third day of incubation. It indicates a weak activity of lecithinase. M-2 strain exhibited gelatinase-negative, nonmotile, but lecithinase-positive phenotype after 48 h of incubation. Lipase activity was negative on egg yolk agar, but for lipase activity detection more accurate approaches, such as the colorimetric method is recommended^45^. Proteolytic activity was additionally detected in both strains. Mentioned above findings indicate that both our *Aeromonas* strains have potential virulent properties. This is particularly true for *A. hydrophila* M-30 which possessed all three major virulence genes associated with enterotoxicity and exhibited activity of some secreted virulence factors.

Aeromonads are well known to be intrinsically resistant to some beta-lactams due to harboring a wide range of chromosomally mediated beta-lactamases of different classes^46^. From a clinical point of view, the most interesting among them is the unique Ambler class B CphA beta-lactamase which is specific only to carbapenems^47^. Several reports showed that the disk-diffusion method failed to detect carbapenem resistance mediated by CphA since a high likelihood of false-negative results^48,49^. Surprisingly, in our study phenotypic resistance to meropenem and imipenem was observed in *A. hydrophila* M-30 strain while *A. encheleia* M-2 had an intermediate category (I) of carbapenem resistance. However, we detected *cphA* gene in both *Aeromonas* strains through PCR with specific primers. These findings confirm the phenotypic resistance profile of these strains to carbapenems. Additionally, we discovered piperacillin-tazobactam resistance in both strains. This phenomenon may be explained by the possible expression of chromosomally encoded *ampC* beta-lactamase gene by *Aeromonas* strains which confer resistance to a wide range of beta-lactams as well as beta-lactamase inhibitors, including tazobactam.

Notably, strain M-30 showed trimethoprim resistant phenotype. Kadlec et al. showed that trimethoprim resistance in *Aeromonas* can be caused by different alleles of *dfrA* or *dfrB* genes, which are frequently associated with class 1 integrons (InC1)^50^. The latter is known to be the most widespread integron class associated with antimicrobial resistance. These mobile genetic elements (MGE) can be embedded in various mobile or conjugative MGE such as plasmids or conjugative transposons which in turn permit its lateral transfer to related or even distantly related bacterial species, which further leads to antimicrobial resistance dissemination^51^. Piotrowska and Popowska showed that genes conferring resistance to aminoglycosides (*aadA1*, *aadA2*) and trimethoprim (*dfrA1*) are the most often found gene cassettes in *Aeromonas* InC1 integrons^52^. However, integrase gene class I (IntI1) as well as typical gene cassettes were not detected in both *Aeromonas* stains, which indicates the absence of integron class I in their genomes. Resistance to trimethoprim in M-30 isolate may be caused by a modified *dfr* allele unrelated to integron class I, although it may be related to other integron classes or mobile elements.

Thus, the detection of *A. encheleia* and *A. hydrophila* in wild rodents potentially supposes a ubiquitous distribution of these bacteria including terrestrial animal organisms. Strains isolated from wild animals demonstrate a resistant phenotype to some beta-lactams as well as trimethoprim. The data we received expanded our understanding of the ecology of these microorganisms and highlight the need for surveillance of these bacteria in wildlife as the major elements in antimicrobial-resistant dissemination in natural ecosystems.

Despite our findings, we recognize the limitations of our work. Animal captures were ​​restricted to a single period; the total number of animal captures was low as well as the total number of isolated Aeromonas strains. We did not conduct any pathological-anatomical investigations and measurements of bacterial load in organs. The exact nature of these strains, whether they were transient or rather resident (harbored in the rodent for a long time), remains vague. More extensive and large-scale research is required to find out whether rodents or other small terrestrial mammals can be a reservoir for bacteria of these species.

## Materials and methods

### Ethics Statement

Animal experiments were performed according to: (I) The Directive 2010/63/EU of the European Parliament and of the Council of 22 September 2010 on the protection of animals used for scientific purposes; (II) The Russian Federation Federal Law No. 498-ФЗ “On Responsible Handling of Animals and on Amending Certain Legislative Acts of the Russian Federation”, adopted on 17 December 2018; (III) ARRIVE 2.0 guidelines^53^. All methods were performed in accordance with the relevant guidelines and regulations.

Fieldwork was carried out on the task of the government assignment by Federal Research Center for Virology and Microbiology. Wild mice were used in this study. None of the rodent species investigated in the present study had protected status. Trapping campaigns were systematically performed with prior explicit agreement from relevant local authorities, and from the owners of the territory where trapping was performed. All procedures involving animals (trapping and killing) were approved by the Ethics Committee of the Federal Research Center for Virology and Microbiology (certificate №: IRECAS_03).

### Samples collection and rodent species

Wild rodents were captured in June 2021 in the Lyskovsky District of the Nizhny Novgorod region, Russia. Animals were captured in the forests on the right bank of the Volga River (56.070217; 45.332745). The soils in this area are loamy and gray forest. The forests are predominantly mixed and varied in the composition of species: oak, birch, linden, maple, hazel, euonymus, mountain ash, as well as various herbs.

Animal carcasses were treated with insecticides and placed in separate containers. Transportation, autopsy, and collection of organs and tissues were carried out on the same day. Identification of species of wild rodents was performed using a guide-identifier of animal species.

### Bacterial strains isolation

The *Aeromonas* strains were isolated from apparently healthy rodents in the framework of a complex study dealing with microbial biodiversity in the natural foci of infections. The captured small rodents were autopsied in aseptic conditions. All procedures were carried out in the biosafety cabinet to get the proper sterility condition and to avoid contamination^54^. The animal's pelt was pretreated with 70% isopropyl alcohol to reduce the microbial load on the surface and reduce the likelihood of contamination. The following organs were aseptically extracted in the corresponding order: lungs, liver, spleen, mesentery, and intestines. The intestines were extracted last to prevent contamination of other internal organs with gut microbiota. Each organ was dipped in 96% ethanol and flamed rapidly through an open flame. The inner part of the organs was obtained via cutting with sterile micro scissors (the intestines were just cut). Extracted inner part of organs was transferred in 10 ml of PSB Broth Base (Himedia, Mumbai, India) and BPW (Himedia, Mumbai, India) to enrichment for 120 h at 22 °C and 24 h at 37 °C respectively. After the enrichment stage, a loopful of each broth PSB and BPW was streaked onto CIN agar (Himedia, Mumbai, India) and XLD agar (Himedia, Mumbai, India) respectively following the incubation for 24 h at 25 °C and 37 °C. Morphologically distinct colonies were plated onto Nutrient agar (Himedia, Mumbai, India) for further cultivation and getting an axenic culture. Received isolates were tested on oxidase activity with OXItest (Erba Lachema) and catalase activity with 3% hydrogen peroxide. All isolates were stored at -80 °C in Nutrient Broth (Himedia, Mumbai, India) containing 15% glycerol.

### 16 s rRNA gene-based strain identification

The 16 s rRNA gene sequencing was performed to identify M-2 and M-30 strains. Bacterial DNA was isolated from overnight grown pure cultures (single colonies were used) through thermal lysis of bacterial suspensions at 95 °C for 15 min. We utilized the following universal primers: 27F: 5’-AGAGTTTGATCMTGGCTCAG-3’ and 1492R: 5’-TACGGYTACCTTGTTACGACTT-3′ ^55^. The amplicons of ~ 1500 bp in length were removed from 1% agarose gel and extracted via an agarose gel DNA extraction kit (Dia-m, Moscow, Russia). Sanger sequencing was performed at the GENOME Center for Collective Use (Moscow, Russia). The consensus of the 16 s rRNA gene was assembled in UGENE (v39.0) software manually^56^. The EzTaxon server was used as a 16 s rRNA reference gene database for identification^57^. Maximum-likelihood phylogeny was reconstructed in MEGA7 software together with 16 s rRNA gene sequences of reference strains from GenBank with 1000 bootstrap replications^58^. The accession numbers of strains used are listed in Table 2.

**Table 2:** The list of *Aeromonas* spp. strains used for phylogenetic analysis.

№	Species	Strain	RefSeq genome accession number
1	*Aeromonas allosaccharophila*	CECT 4199	GCF_000819685.1
2	*Aeromonas allosaccharophila*	FDAARGOS_933	GCF_016026615.1
3	*Aeromonas aquatica*	AE235	GCF_000764655.1
4	*Aeromonas australiensis*	CECT 8023	GCF_000819725.1
5	*Aeromonas australiensis*	Aer294	GCF_021538905.1
6	*Aeromonas bestiarum*	CBA100	GCF_000746985.1
7	*Aeromonas bestiarum*	GA97-22	GCF_002906925.1
8	*Aeromonas caviae*	YL12	GCF_000721855.1
9	*Aeromonas caviae*	WP8-S18-ESBL-04	GCF_014169735.1
10	*Aeromonas dhakensis*	SSU	GCF_000298055.1
11	*Aeromonas dhakensis*	71,431	GCF_020405345.1
12	*Aeromonas encheleia*	NCTC12917	GCF_900637545.1
13	*Aeromonas enteropelogenes*	1999lcr	GCF_000687355.2
14	*Aeromonas enteropelogenes*	FDAARGOS_1537	GCF_020341435.1
15	*Aeromonas finlandensis*	4287D	GCF_000764645.1
16	*Aeromonas hydrophila*	NF2	GCF_000708065.1
17	*Aeromonas hydrophila*	OnP3.1	GCF_017310215.1
18	*Aeromonas jandaei*	FDAARGOS_986	GCF_016127195.1
19	*Aeromonas lacus*	AE122	GCF_000764665.1
20	*Aeromonas media*	ARB13	GCF_000754915.1
21	*Aeromonas media*	TR3_1	GCF_020423125.1
22	*Aeromonas piscicola*	LMG 24,783	GCF_000820005.1
23	*Aeromonas popoffii*	CIP 105,493	GCF_000820025.1
24	*Aeromonas popoffii*	ID682	GCF_018138755.1
25	*Aeromonas rivipollensis*	KN-Mc-11N1	GCF_003015165.1
26	*Aeromonas rivipollensis*	G78	GCF_010974825.1
27	*Aeromonas salmonicida*	Y567	GCF_001466435.1
28	*Aeromonas salmonicida*	SRW-OG1	GCF_012931585.1
29	*Aeromonas sanarellii*	LMG 24,682	GCF_000820085.1
30	*Aeromonas sanarellii*	NS1	GCF_019334425.1
31	*Aeromonas sobria*	CECT 4245	GCF_000820145.1
32	*Aeromonas sobria*	JF2635	GCF_002843345.1
33	*Aeromonas tecta*	CECT 7082	GCF_000820185.1
34	*Aeromonas veronii*	CIP 107,763	GCF_000820285.1
35	*Aeromonas veronii*	FDAARGOS_632	GCF_008693705.1

### Multilocus sequence analysis (MLSA)-based strains identification

Because 16 s rRNA gene-based identification of *Aeromonas* species has not enough discriminatory power we used multilocus sequence analysis (MLSA), also known as multilocus phylogenetic analysis (MLPA) as a more sophisticated method appropriate to resolve the complex taxonomy of Aeromonads. Loci from the *Aeromonas* MLST scheme suggested by Martino et al. were used^59^. A set of 5 loci including *groL*, *gltA*, *metG*, *ppsA*, and *recA* was chosen. Primers as well as cycling conditions suggested by the original scheme mentioned above were used. The final volume of PCR mixture was 25 µl with 1 µl of template DNA, 1 µl of each 10 µM primer, 2,5 µl 10X of Encyclo buffer (Evrogen, Moscow, Russia), 0,5 µl 10 µM of dNTP, 0,5 µl of Encyclo polymerase (Evrogen, Moscow, Russia), and 18,5 µl of deionized nuclease-free water. The amplification conditions were as follows: an initial step at 94 °C for 2 min; 35 cycles of denaturation at 94 °C for 10 s, annealing at different temperatures (*groL* 56 °C, *gltA* 58 °C, *metG* 57 °C, *ppsA* 60 °C, and *recA* 57 °C) for 30 s, and extension at 72 °C for 2 min. Amplicons were detected in 1% agarose gel through horizontal electrophoresis. The target amplicons were extracted from the gel and purified with the GeneJET Gel Extraction kit (Dia-m, Moscow, Russia). Sanger sequencing was performed at the GENOME Center for Collective Use (Moscow, Russia), which is equipped with an Applied Biosystems 3730 DNA Analyzer. Obtained DNA sequences from 5 loci were concatenated and utilized for further phylogenetic analysis. GenBank was used to receive loci from genomes of other relative species of the *Aeromonas* genus for phylogeny reconstruction (Table 1). Maximum-likelihood phylogeny was reconstructed in MEGA7 software with 1000 bootstrap replications^58^.

### Antimicrobial susceptibility testing

Susceptibility to the antimicrobial agents was tested by the disk-diffusion method following the Clinical and Laboratory Standards Institute (CLSI) guidance on Mueller–Hinton agar (Himedia, India, Mumbai). In total antimicrobial susceptibility testing to 13 commonly used antimicrobials was performed. The following antibiotics were used: piperacillin-tazobactam (30–6 µg), cefepime (30 µg), ceftazidime (10 µg), aztreonam (30 µg), imipenem (10 µg), meropenem (10 µg), amikacin (10 µg), gentamicin (10 µg), tetracycline (30 µg),ciprofloxacin (5 µg), levofloxacin (5 µg), enrofloxacin (5 µg), trimetoprim-sulfametoxazol (1.25–23.75 µg), chloramphenicol (30 µg), trimethoprim (5 µg). The *Escherichia coli* strain ATCC 25,922 was utilized as a control.

### Polymerase chain reaction (PCR) assays

*Aeromonas* strains were cultured in Tryptone Soya Broth (Himedia) at 37 °C for 18–20 h. Cells were harvested and total DNA was extracted through thermal lysis of bacterial cells suspension at 95 °C for 15 min. DNA of each strain was utilized as a template to detect antimicrobial resistance determinants, including *cphA* class B beta-lactamase gene^60^, class I integrase gene (*intI1*)^61^, and variable integron region (gene cassettes)^62^. Additionally, the main aeromonads virulence genes (genes associated with enterotoxigenicity) were screened, including the hemolysin *hlyA* gene^63^, cytotonic heat-labile, and heat-stable enterotoxin genes (*alt* and *ast* respectively)^64^.

The PCR cycling conditions used were as the following: initial denaturation at 94 °C for 3 min, then 30 cycles of denaturation at 94 °C for 30 s, annealing depending on primers used, extension at 72 °C for 1 min, and a final extension at 72 °C for 5 min. The primers utilized for amplification, the expected sizes of the amplification products as well as annealing temperature are listed in Table 3. Amplicons were visualized via 1% agarose gel electrophoresis. Sanger sequencing of *intI1* gene and *in* (variable regions) locus was performed at the GENOME Center for Collective Use (Moscow, Russia).

**Table 3:** Primers used for PCR analysis.

Primer	Target gene	Nucleotide sequence (5′ → 3′)	Amplicon size (bp)	Annealing T, °C
CphA-F	*cphA*	TGGTGCTGRTGGCGAGTT	346	56
CphA-R		GCCCARTCGCTCTTCATCA		
IntI1-F	*intI1*	GTTCGGTCAAGGTTCTG	920	50
IntI1-R		GCCAACTTTCAGCACATG		
InF	Variable region	GGCATACAAGCAGCAAGC	Variable	50
InB		AAGCAGACTTGACCTGAT		
hlyA-F	*hlyA*	CCACGCAAATTCATCACG	1 079	55
hlyA-R		ATCCTTGTTCACCTCGAC		
alt-F	*alt*	TGACCCAGTCCTGGCACGGC	442	55
alt-R		GGTGATCGATCACCACCAGC		
ast-F	*ast*	TCTCCATGCTTCCCTTCCACT	331	55
ast-R		GTGTAGGGATTGAAGAAGCCG		

### Hemolysis assay

Columbia Agar Base (Himedia, Mumbai, India) supplemented with 5% (v-v) of mechanically defibrinated human O-group blood was utilized to check bacterial hemolytic activity (a blood sample was aseptically collected from a healthy adult volunteer with his documentary consent). Both overnight *Aeromonas* cultures were plated onto blood agar with further incubation for 24 h and 48 h at 3 different temperature points: 25 °C, 35 °C, and 37 °C. Clear transparent zones around the grown colonies were interpreted as beta-hemolysis, color change (to greenish or yellow-greenish) was interpreted as alpha-hemolysis, and the absence of any visible changes was interpreted as gamma-hemolysis.

### Phenotypic assessment of some virulence factors production

The culture-based method was implemented to evaluate the secretion of virulence factors. The following tests were carried out: gelatin hydrolysis, lecithinase, lipase activity test, and motility test. Mentioned above-secreted virulence factors are known to be involved in invasion by direct damage of host tissue, inactivation of the complement system, cytotoxicity, etc^65^.

Bacterial strains were grown previously at 37 °C for 24 h in Tryptone Soya Broth (Himedia). The Gelatin hydrolysis test was performed with a Micro-Gelatinase-NICF kit (RCP, Saint-Petersburg, Russia) following manufacturer instructions. Briefly, the gelatin deep tubes were inoculated with 24 h bacterial cultures. Test tubes were incubated at 37 °C for 72 h. As a negative control, noninoculated gelatin deep tubes were used. After incubation tubes were cooled at 4–5 °C for 30 min. Visible hydrolysis (liquid fraction formation) was considered a positive result. For lecithinase and lipase activity detection egg yolk agar base (Himedia) with 2.5% yolk was utilized, and incubation was carried out for 18–48 h and continued for 7 days (lipase activity may be delayed). The positive reaction was considered if an opaque (visible precipitation) was formed around the bacterial colonies for lecithinase activity; a clear zone formation for protease activity, and forming an iridescent sheen on the surface of the colonies for lipase activity. The motility test was performed via inoculation of the Motility Test Medium (Himedia) and following incubation at 37 °C. Motility was considered positive if the diffuse zone was formed of growth flaring out from the inoculation line.

## Supplementary Information

Supplementary Information 1.

Supplementary Information 2.

Supplementary Information 3.

Supplementary Information 4.

Supplementary Information 5.

Supplementary Information 6.

## Data Availability

The data sets generated during the current study are available in the GenBank repository: partial 16 s rRNA gene sequence of *Aeromonas encheleia* M-2 (accession no. OP847777) and *Aeromonas hydrophila* (accession no. OP847778).
