# Supplementary figures and images for: The first detection of two Aeromonas strains in mice of the genus Apodemus
Source: Sci Rep. 2023 Mar 15;13:4315. doi: 10.1038/s41598-023-31306-3 (PMC10017686; doi:10.1038/s41598-023-31306-3)

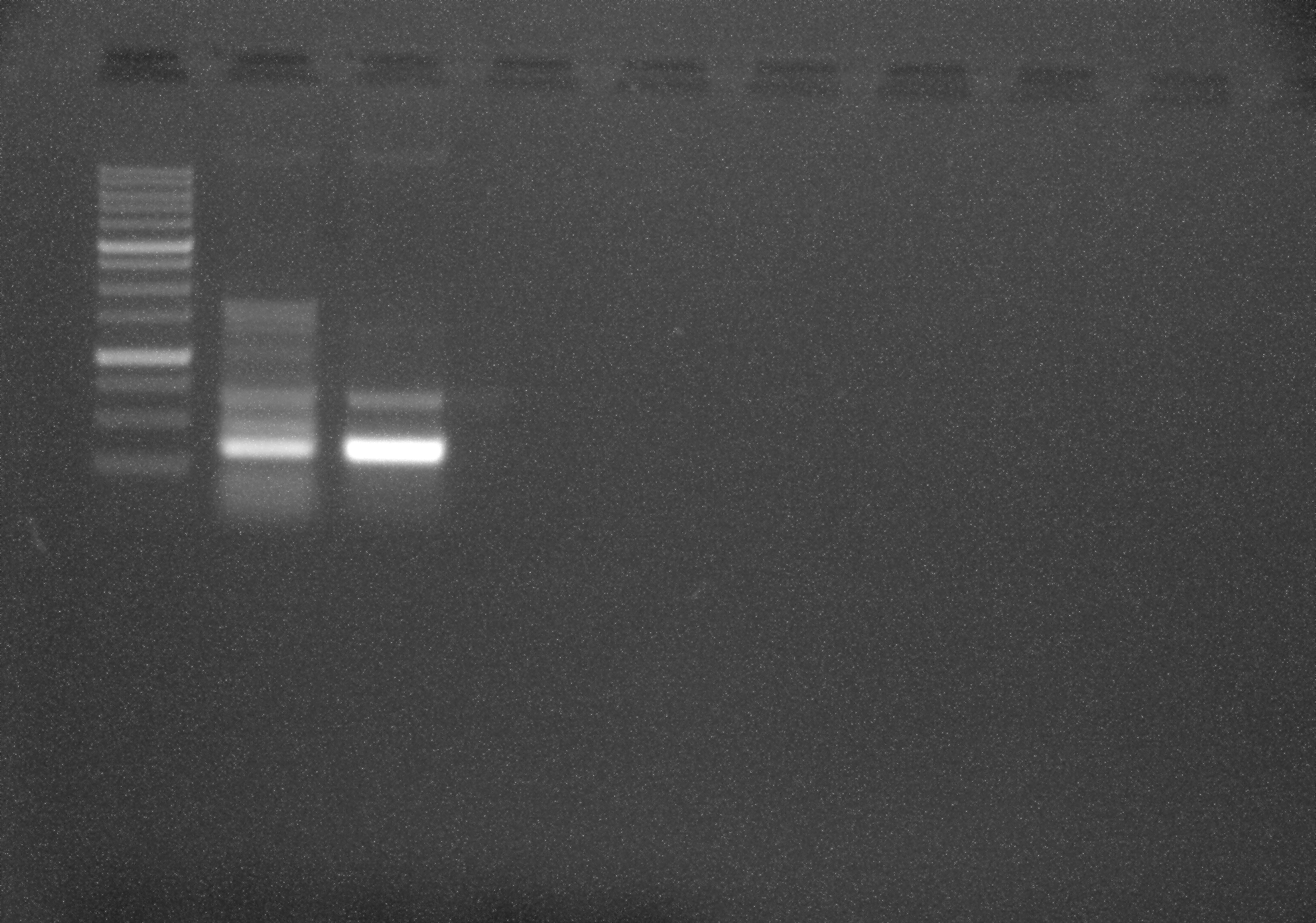

Supplement: Supplementary file 1 — Supplementary Information 1. [file 41598_2023_31306_MOESM1_ESM.png]

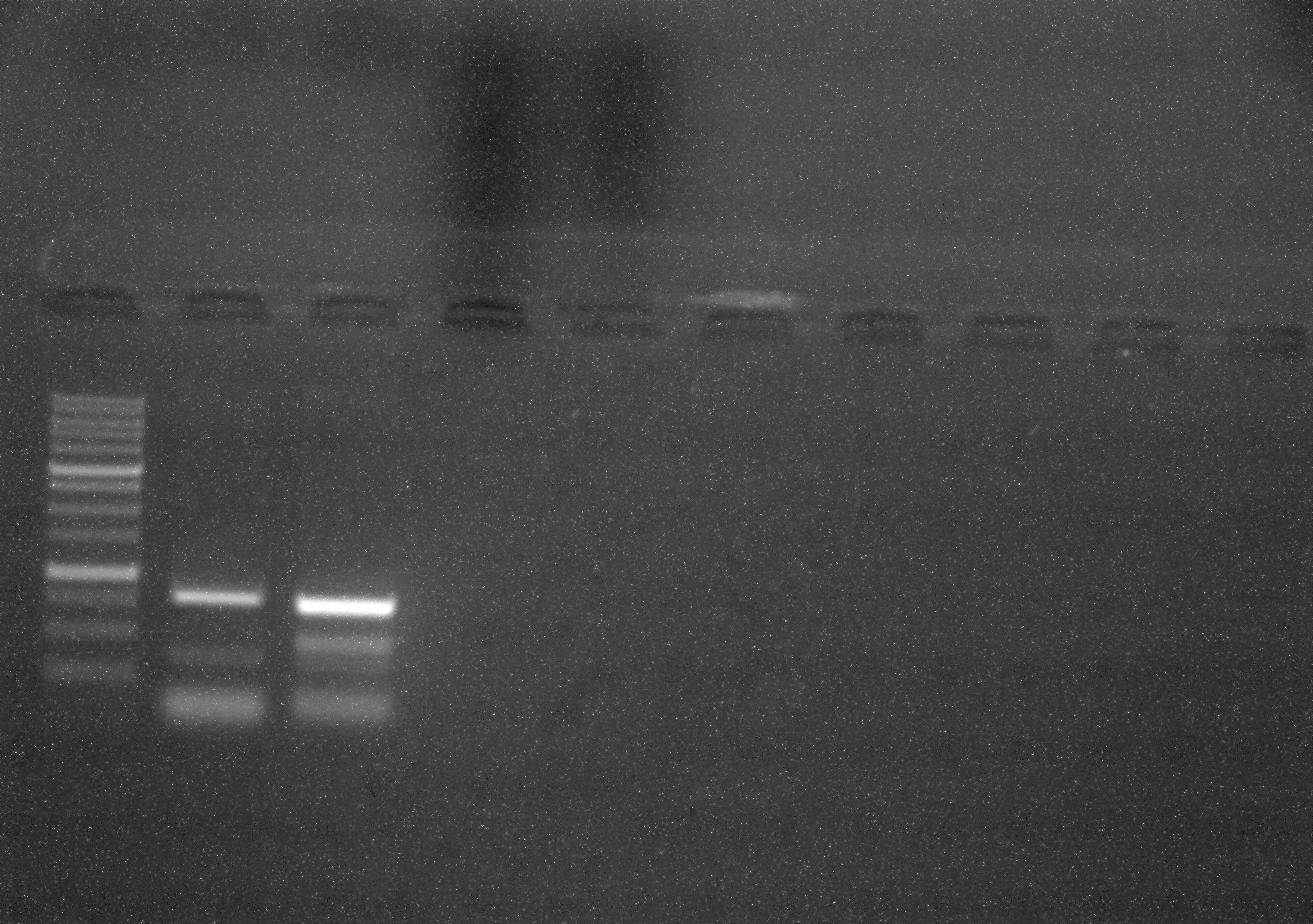

Supplement: Supplementary file 2 — Supplementary Information 2. [file 41598_2023_31306_MOESM2_ESM.png]

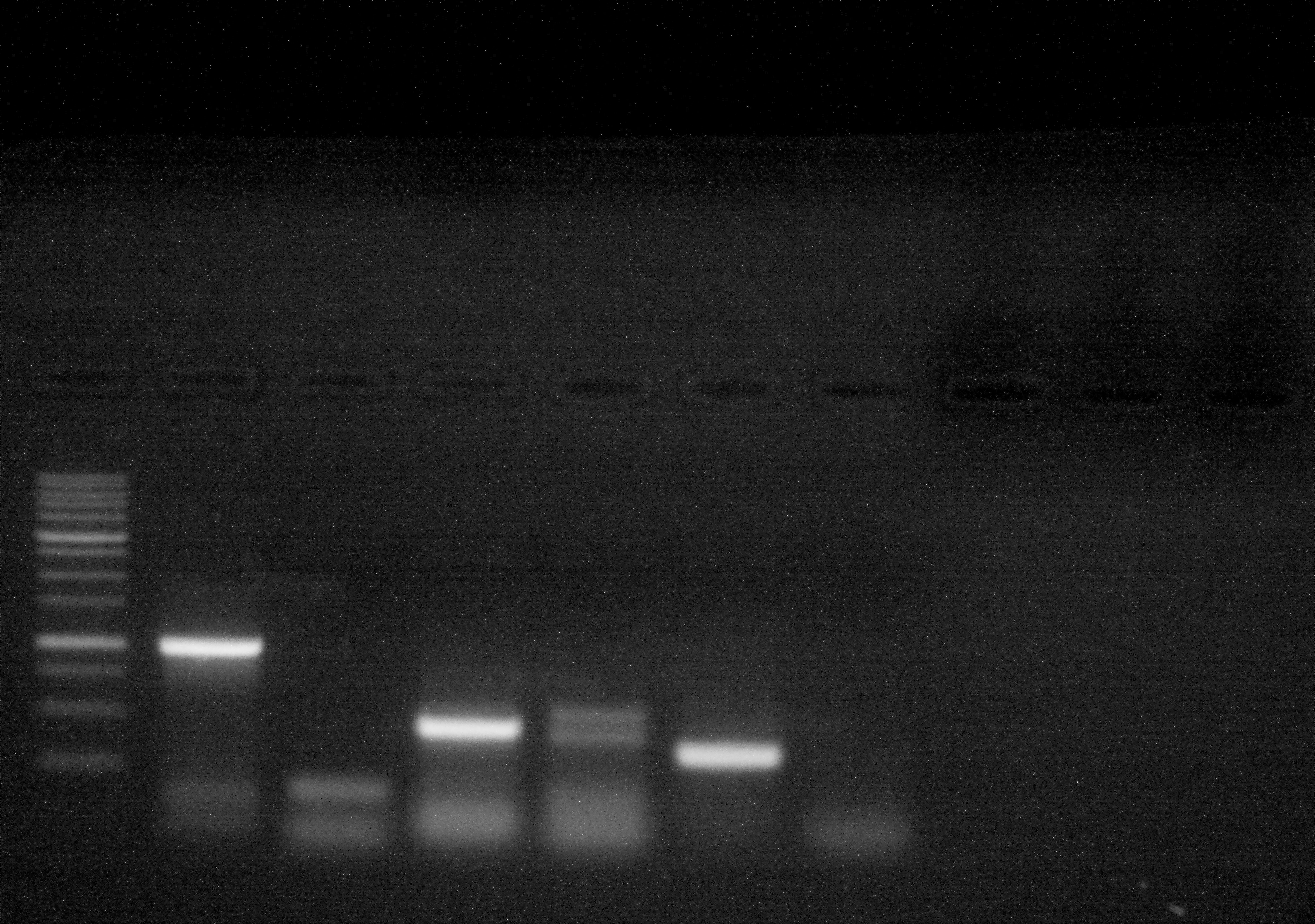

Supplement: Supplementary file 3 — Supplementary Information 3. [file 41598_2023_31306_MOESM3_ESM.png]

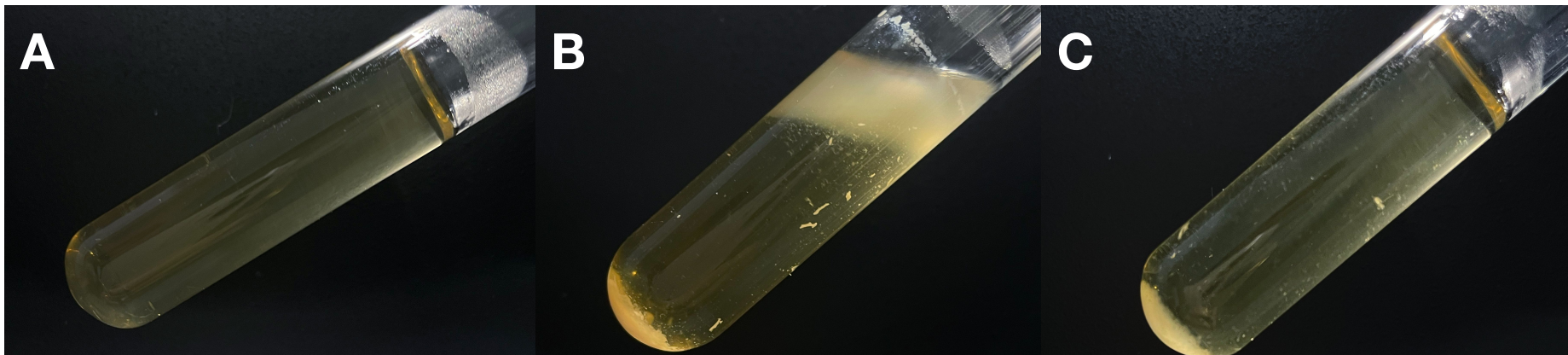

**Figure S4.** Results of gelatinase test:

**A** - negative control;

**B** - *A. hydrophyla* M-30;

**C** - *A. encheleia* M-2;

Supplement: Supplementary file 4 — Supplementary Information 4. [file 41598_2023_31306_MOESM4_ESM.pdf]

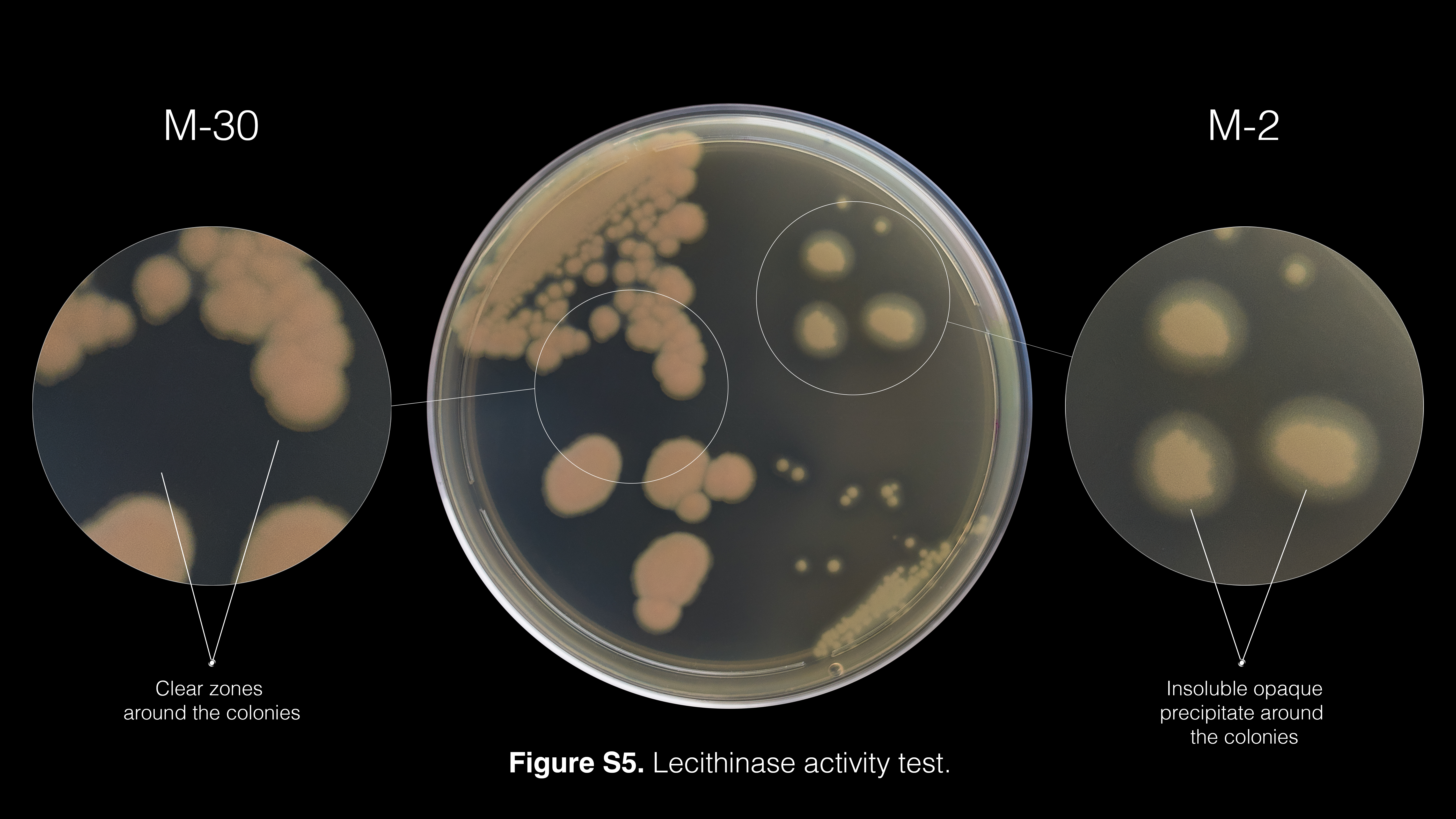

Supplement: Supplementary file 5 — Supplementary Information 5. [file 41598_2023_31306_MOESM5_ESM.jpg]

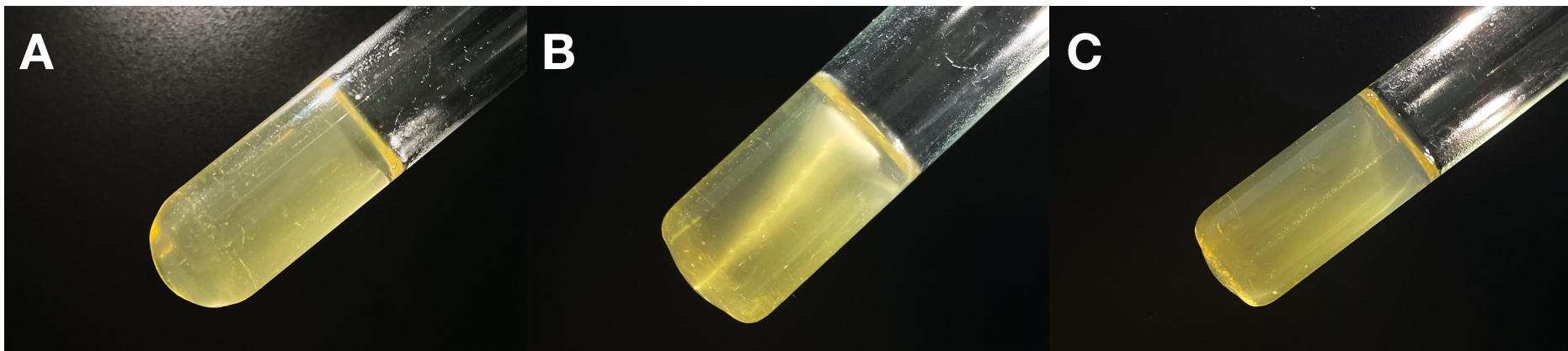

**Figure S6.** Results of motility test:

**A** - negative control;

**B** - *A. hydrophyla* M-30;

**C** - *A. encheleia* M-2;

Supplement: Supplementary file 6 — Supplementary Information 6. [file 41598_2023_31306_MOESM6_ESM.pdf]
